# A Meta-analysis of Voxel-based Brain Morphometry Studies in Obstructive Sleep Apnea

**DOI:** 10.1038/s41598-017-09319-6

**Published:** 2017-08-30

**Authors:** Yan Shi, Lizhou Chen, Taolin Chen, Lei Li, Jing Dai, Su Lui, Xiaoqi Huang, John A. Sweeney, Qiyong Gong

**Affiliations:** 1 Huaxi MR Research Center (HMRRC), Department of Radiology, West China Hospital of Sichuan University, Chengdu, Sichuan 610041 China; 2 Department of Sociology and Psychology, School of Public Administration, Sichuan University, Chengdu, Sichuan 610041 China; 3 Huaxi MR Research Center, Department of Psychiatry, West China Hospital of Sichuan University, Chengdu, Sichuan 610041 China; 4 Department of Psychiatry and Behavioral Neuroscience, University of Cincinnati, Cincinnati, Ohio USA; 5 Department of Psychology, Chengdu Normal University, Chengdu, Sichuan 611130 China; 6 Department of Psychoradiology, Chengdu Mental Health Center, Chengdu, Sichuan 610031 China

## Abstract

Gray matter (GM) anomalies may represent a critical pathology underlying obstructive sleep apnea (OSA). However, the evidence regarding their clinical relevance is inconsistent. We conducted a meta-analysis of voxel-based morphometry (VBM) studies of patients with OSA to identify their brain abnormalities. A systematic search was conducted based on PRISMA guidelines, and a meta-analysis was performed using the anisotropic effect-size-based algorithms (ASE-SDM) to quantitatively estimate regional GM changes in patients with OSA. Fifteen studies with 16 datasets comprising 353 untreated patients with OSA and 444 healthy controls were included. Our results revealed GM reductions in the bilateral anterior cingulate/paracingulate gyri (ACG/ApCG), left cerebellum (lobules IV/V and VIII), bilateral superior frontal gyrus (SFG, medial rostral part), right middle temporal gyrus (MTG), and right premotor cortex. Moreover, GM reductions in the bilateral ACG/ApCG were positively associated with body mass index (BMI) and age among patients with OSA, and GM reductions in the SFG (medial rostral part) were negatively associated with Epworth sleepiness scale (ESS) scores and sex (male). These abnormalities may represent structural brain underpinnings of neurocognitive abnormalities and respiratory-related abnormalities in OSA. ﻿In particular, this study adds to Psychoradiology, which is a promising subspecialty of clinical radiology mainly for psychiatric disorders.

## Introduction

Obstructive sleep apnea (OSA) involves sleep-induced apnea with complete or partial obstruction of the upper airway during sleep, leading to reduced brain oxygenation, transient arousal from sleep and sleep fragmentation. OSA affects approximately 9% of middle-aged men and 4% of middle-aged women^1^; estimates for the middle-aged to elderly population range from 49.7% to 79.2% in men and 23.4% to 54.3% in women^2^. OSA is a growing health concern because it has many unfavorable consequences, including daytime drowsiness and associated cardiovascular diseases, cognitive impairment (involving memory, attention and vigilance^3, 4^), and emotional problems, including depression^5^. OSA may be related to increased risk of neurodegenerative conditions, such as Alzheimer’s disease^6^.

In most epidemiological studies, the definition of OSA is based on the apnea-hypopnea index score (AHI) (the number of obstructive respiratory events per hour during sleep), which reflects the degree of departure from the normal physiology of breathing during sleep. OSA is defined as AHI ≥ 5 (mild = 5–15; moderate = 15–30; severe > 30) with associated symptoms (daytime sleepiness, cognitive impairments)^7^, although some studies have based OSA diagnoses on the respiratory disturbance index (RDI). AHI is defined by the American Academy of Sleep Medicine (AASM) as a ≥ 50% reduction in airflow for 10 seconds or longer or a clear reduction (but < 50%) with either an arousal or ≥ 3% (or ≥ 4%) oxygen desaturation, and RDI is defined as AHI with or without respiratory effort-related arousals (RERAs)^8^. In addition to AHI and RDI, body mass index (BMI), the Epworth sleepiness scale (ESS) and the oxyhemoglobin desaturation index (ODI) are frequently used to characterize patients with OSA. BMI is an indicator of body mass, and obesity (BMI ≥ 30) increases the risk of OSA^9^. The ESS is a self-administered questionnaire used to subjectively measure daytime sleepiness^10^. A score ≥ 10 is considered to represent excessive daytime sleepiness (EDS)^11, 12^. The ODI is calculated as the number of times per hour that the blood’s oxygen desaturation is ≥ 3% or ≥ 4% from baseline during sleep^8^. Significant correlations between ODI and BMI^13^ and between AHI and ODI^14^ have been reported, but the correlation between AHI and ODI is diminished among obese patients, indicating that oximetry may be more important in obese than in normal-weight patients^15^.

During the past three decades, developments in neuroimaging using MRI have come to provide highly sensitive, noninvasive approaches for investigating the neural mechanisms of brain alterations associated with various medical disorders, such as OSA. Although the exact pathology of OSA remains unclear, both hypoxemia and sleep fragmentation contribute to the neural deficits related to this disease^16^. To date, structural neuroimaging studies using voxel-based morphometry (VBM) have demonstrated widespread gray matter (GM) deficits in patients with OSA in multiple neocortical regions (frontal, prefrontal, ventral medial prefrontal, occipital, lateral temporal and parietal cortices), limbic regions (anterior/mid-cingulate gyrus, insula, amygdala, hippocampus/parahippocampal gyrus), basal ganglia, and cerebellum^17–19^. Researchers have also explored the relationships between structural brain abnormalities and clinical signs and symptoms of OSA. Celle *et al*.^20^ reported that AHI and ODI scores were inversely correlated with the GM volume in the bulbopontine area and cerebellum in patients with OSA. However, the localization of deficits varies considerably across studies, and some results are conflicting. For example, the insula has been reported to show both significant GM atrophy^18, 21^ and hypertrophy^22, 23^. The reasons accounting for these discrepancies include demographic (e.g., age and sex) and methodological factors (e.g., statistical thresholds and smaller patient cohorts with variability in the chronic and acute severity of OSA).

Reviews and meta-analyses have been conducted to evaluate these results and identify robust conclusions across studies. Regarding review articles, Zimmerman & Aloia^24^ found that the hippocampus may be a common atrophic region, and Huynh *et al*.^19^ suggested that there were few robust differences between OSA patients and healthy controls. Celle *et al*.^25^ stated that measuring GM volume changes in patients with OSA would not yield valid results unless large cohorts were studied to permit statistical control for various clinical and demographic confounding influences. In addition to these qualitative observations, coordinate-based meta-analyses have been used to facilitate quantitative analyses combining data from different studies. The first VBM meta-analysis, conducted by Weng *et al*.^26^ utilizing ALE software, evaluated 8 VBM studies^16, 21, 27–32^ containing 213 patients and 195 controls. They found that patients with OSA exhibited significant GM reductions in the bilateral parahippocampus and frontotemporal regions (although the evidence of the latter was less convincing). Recently, Tahmasian *et al*.^33^ published another coordinate-based meta-analysis using ALE software. In their study, VBM and functional magnetic resonance imaging (fMRI) studies were combined (4 VBM studies^21, 30–32^, 9 fMRI studies^34–42^, and 2 VBM & fMRI studies^22, 43^), which contained 290 OSA patients and 290 controls. The authors reported significant structural atrophy and functional disturbances in the right amygdala/hippocampus and right insula.

The previous two meta-analyses have provided useful information concerning OSA. However, there were important limitations. First, the VBM literature on OSA has grown substantially since the first VBM meta-analysis was published. Thus, a new meta-analysis including more VBM studies should provide more precise results. Although it included updated literature, the meta-analysis conducted by Tahmasian *et al*.^33^ explored only convergent brain changes on VBM and resting-state fMRI studies. Such a combination might fail to identify all regions with anatomic alterations. Second, the meta-analytic tool used by previous studies did not consider the effects of important demographic and clinical factors. One newer coordinate-based meta-analytic tool, the anisotropic effect-size-based algorithm (AES-SDM), enables such exploration and allows subgroup and sensitivity analyses to establish the robustness of the results. Third, although both software platforms are coordinate-based, there are some inherent differences between them, e.g., SDM takes the *p* value of each coordinate into account, while ALE does not.

Therefore, we conducted a meta-analysis using AES-SDM software to identify the most prominent and replicable GM changes among patients with OSA. We performed an updated literature search, and a sensitivity analysis was applied to evaluate the robustness of the results. Second, we examined clinical and demographic characteristics of the patient groups in the studies included in our meta-analysis, and we performed a meta-regression analysis to explore the associations between these features and identified brain alterations.

## Methods

### Inclusion of studies

**Figure 1:**
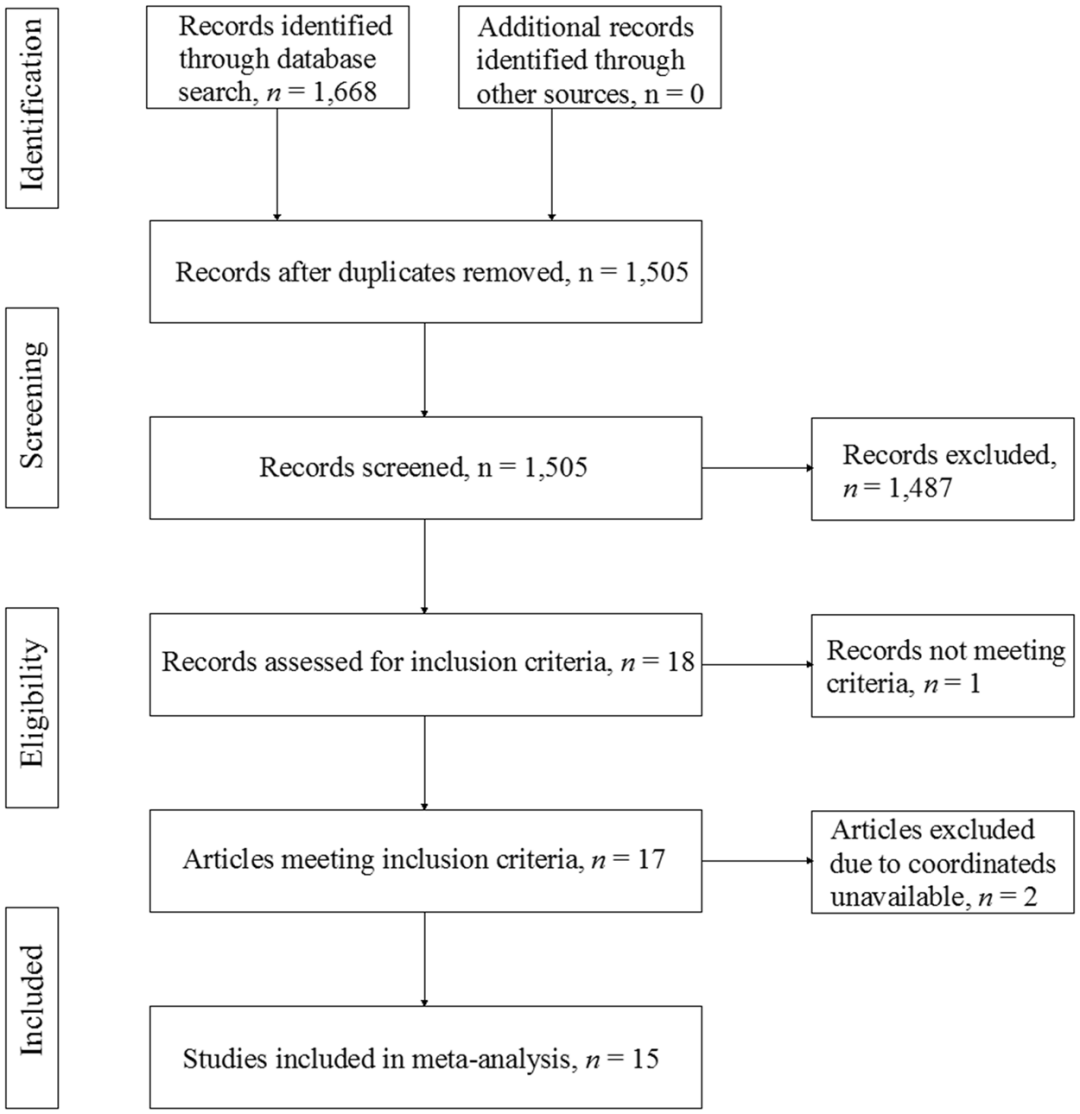
PRISMA flowchart for the meta-analysis of voxel-based morphometry studies in patients with obstructive sleep apnea.

We searched the PubMed, Science Direct, Medline, Embase and Web of Science databases for literature from January 2000 to December 2016 using the keywords (“obstructive sleep apnea” OR “sleep apnea” OR “sleep disordered breathing” OR “sleep related breathing disorders” OR “OSA”) and (“voxel” OR “morphometry” OR “voxel-based morphometry” OR “VBM”). Furthermore, we checked the reference list of each study to identify additional studies for inclusion. The following were criteria for inclusion: (1) voxel-based morphometry was applied to analyze whole-brain gray matter volume (GMV) or gray matter concentration (GMC) or gray matter density (GMD) in patients with OSA (since both GMC and GMD reflect GM volumetric changes, we uniformly use GMD in the following text); (2) untreated patients with OSA were compared with healthy control subjects; and (3) articles reported whole-brain GM alterations in Montreal Neurological Institute (MNI) or Talairach stereotactic space coordinates (x, y, z). We followed the PRISMA guidelines to identify studies^44^ as illustrated in the PRISMA flowchart in Fig. 1.

### VBM meta-analysis

Prior to conducting the meta-analysis, the reported peak coordinates of regional alterations were extracted to construct an analysis file. We also recorded the following variables from each study: sample size, percentage of male subjects, mean age of subjects, clinical characteristics of the patient group (BMI, AHI, ODI, and ESS), and method utilized to correct whole-brain results for multiple comparisons.

The analytical processes used in the meta-analysis are described in the AES-SDM tutorial (http://sdmproject.com/software/Tutorial.pdf) and related publications. As an intrinsic whole-brain analysis technique, AES-SDM does not restrict analyses to *a priori* regions of interest, thereby providing a regionally unbiased assessment of neuroanatomical differences on a point-by-point basis across the entire brain^45^. Because it can make use of the reported peak coordinates to recreate maps of the effect size of group differences, this method has been widely applied in many meta-analytic studies of neuropsychiatric disorders^46, 47^. Because this method has been described in detail elsewhere^48^, we describe below the primary analysis steps in brief. First, we ensured that the extracted coordinates from each study utilized the same threshold throughout the whole brain to avoid biases toward liberally thresholded brain regions. In cases in which the results were reported as both uncorrected and corrected for multiple comparisons, we analyzed the corrected results. Second, an effect-size signed map of the patient-control differences in GM was recreated for each study independently. Third, the mean map was generated by voxel-based calculation of the mean of all study maps and weighted by the inverse of each study variance to account for inter-study heterogeneity. We used the standard threshold (uncorrected *p* < 0.005, extent threshold of clusters > 10 voxels) recommended by the AES-SDM software developer, which was proposed to optimally balance the sensitivity and specificity of results. Other parameters included the following: anisotropy = 1.0; isotropic full-width at half-maximum (FWHM) = 20 mm; and peak height threshold = 1. MRIcron software (http://www.cabiatl.com/mricro/mricron/) was used to visualize AES-SDM maps by overlaying them onto a high-resolution brain template generated by the International Consortium for Brain Mapping.

### Sensitivity analysis

A whole-brain jack-knife sensitivity analysis was used to test the replicability of the results using the same threshold as the pooled meta-analysis. We repeated the main analysis *n* times (*n* = the number of total datasets), each time discarding a different study, to determine whether the results of the pooled meta-analysis remained significant.

### Subgroup analysis

We initially planned to conduct three subgroup analyses: male-only patient group vs. female-included patient group; middle-aged group (mean age = 40–60 years) vs. old-age group (mean age ≥ 60 years); and EDS group vs. non-EDS group. However, since the numbers of studies including females and elderly/non-EDS studies were insufficient to obtain reliable results, analyses of these subgroups were precluded.

### Meta-regression analysis

Several relevant demographic and clinical factors, such as the percentage of male patients and the mean age, BMI, AHI, ODI, and ESS of patients, were applied in the regression analysis to explore their association with GM changes. To minimize the detection of spurious relationships, we set the probability threshold to *p* < 0.0005 in these analyses, as recommended by the software developer. The regression needed to be detected both in the slope and in one of the extremes of the regressors, and analyses were restricted to brain regions identified in the primary meta-analysis comparing patients and controls^48^.

## Results

The search strategy initially identified 1,668 records; after screening, 17 studies^16, 18–23, 27–32, 43, 49–51^ met our inclusion criteria. Two additional studies were excluded because their coordinates were unavailable^27, 51^. One of the included studies, which was conducted by Huynh *et al*.^19^, separated the OSA patients into two groups: patients treated with a sham continuous positive airway pressure (CPAP) device and those treated with an active CPAP device. The authors separately compared the two groups of patients at baseline (i.e., before treatment) with the same healthy control group; thus, the respective resultant coordinates were included as separate datasets in our study. Therefore, 15 studies with 16 datasets containing 353 patients with OSA (321 males and 32 females; mean age, 49.3 years) and 444 healthy controls (330 males and 114 females; mean age, 52.0 years) were included in our meta-analysis. Each study was reviewed by at least 2 authors to obtain agreement on its clinical and demographic aspects, as summarized in Table 1. Between the OSA group and healthy controls, we detected no significant difference in mean age, and there was a sex ratio difference between patients with OSA and healthy controls in only one study^50^.

**Table 1:** Demographic and clinical characteristics of subjects in the 16 voxel-based morphometry datasets included in the meta-analysis.

Study	Subjects, n (male, %)		Mean age (SD), years		Clinical characteristics of OSA group, mean (SD)				Threshold	Main findings
	**OSA**	**HC**	**OSA**	**HC**	**BMI**	**AHI**	**ODI**	**ESS**		
Morrell *et al*.^49^	7 (100)	7 (100)	50 (NA)	NA	NA	28 (NA)	NA	14 (NA)	SVC corrected, *p* < 0.05	GMD↓ in left hippocampus
O’Donoghue *et al*.^28^^*^	27 (100)	24 (100)	45.7 (10.10)	43.3 (9.40)	33.2 (4.70)	71.7 (17.00)	NA	13.1 (3.90)	FDR corrected, *p* < 0.05	no significant differences
Morrell and Twigg^29^	22 (100)	17 (100)	51.8 (15.4)	53.1 (14.00)	32.4 (5.60)	53.1 (14.00)	NA	13.3 (4.20)	uncorrected, *p* < 0.05	GMD↓ in bilateral parahippocampus
Yaouhi *et al*.^30^	16 (93.75)	14 (92.86)	54.8 (5.71)	52.71 (7.01)	NA	38.31 (14.33)	35 (13.53)	12.5 (4.50)	cluster-wise corrected, *p* < 0.05	GMD↓ in bilateral prefrontal cortex, bilateral inferior parietal gyrus, right temporal cortex, occipital cortex, right thalamus, some basal ganglia regions, right hippocampus/parahippocampal gyrus and cerebellum
Celle *et al*.^20^	25 (44)	127 (34.65)	66 (0.80)	66 (0.60)	27.3 (3.20)	39.5 (11.50)	24.2 (9.00)	6 (4.60)	cluster-wise corrected, *p* < 0.05	GMV↓ in brainstem and cerebellum
Joo *et al*.^21^	36 (100)	31 (100)	44.7 (6.70)	44.8 (5.40)	26 (2.70)	52.5 (21.70)	NA	10.4 (3.70)	FDR corrected, *p* < 0.05	GMD↓ in left gyrus rectus, bilateral superior frontal gyri, left precentral gyrus, bilateral frontomarginal gyri, bilateral anterior cingulate gyri, right insular gyrus, bilateral caudate nuclei, bilateral thalami, bilateral amygdalo-hippocampi, bilateral inferior temporal gyri, and bilateral quadrangular and biventer lobules in the cerebellum
Morrell *et al*.^31^	60 (95)	60 (91.67)	47.3 (NA)	46.1 (NA)	32 (NA)	55 (NA)	33 (NA)	13.2 (NA)	FDR corrected, *p* < 0.05	GMV↓ in right middle temporal gyrus and left cerebellum
Canessa *et al*.^16^	17 (100)	15 (100)	44 (7.63)	42.15 (6.64)	31.24 (4.35)	55.83 (19.08)	NA	11.94 (5.47)	FWE corrected, *p* < 0.05	GMV↓ in left hippocampus (entorhinal cortex), left posterior parietal cortex and right superior frontal gyrus
Torelli *et al*.^32^	16 (81.25)	14 (64.29)	55.8 (6.70)	57.6 (5.20)	31.7 (4.40)	52.5 (26.00)	51 (23.30)	8.5 (4.50)	FWE corrected, *p* < 0.05	GMV↓ in right hippocampus
Zhang *et al*.^43^	24 (100)	21 (100)	44.6 (7.4)	40.6 (11.40)	29.8 (4.40)	54.7 (19.90)	NA	15.2 (7.30)	FWE corrected, *p* < 0.05	GMV↓ in left medial prefrontal cortex and left posterior inferior frontal gyrus
Fatouleh *et al*.^22^	17 (88.24)	15 (80.00)	55 (3.00)	53 (3.00)	31 (2.00)	36 (4.00)	NA	9 (1.00)	FDR corrected, *p* < 0.05	GMV↑ in medulla/pons/cerebellum, bilateral insula, bilateral primary motor cortex, left hippocampus, left premotor cortex
Huynh *et al*.^19^ ^a^	13 (100)	7 (100)	44 (2.00)	41.4 (3.10)	26.1 (0.60)	34 (4.80)	NA	NA	FDR corrected, *p* < 0.055	no significant differences
Huynh *et al*.^19^ ^b^	14 (100)	7 (100)	42.9 (2.2)	41.4 (3.1)	28.7 (1.1)	43.4 (5.90)	NA	NA	FDR corrected, *p* < 0.05	no significant differences
Innes *et al*.^50^	19 (68.42)	19 (31.58)	56.7 (7.8)	50 (9.7)	29.5 (NA)	18.5 (NA)	NA	6.4 (NA)	FDR corrected, *p* < 0.05	no significant differences
Kim *et al*.^18^	21 (100)	59 (100)	49.8 (7.7)	44.3 (10.1)	27.4 (3.8)	60.4 (23.50)	NA	13.9 (4.80)	FDR corrected, *p* < 0.05	GMV↓ in bilateral lateral prefrontal, central, and anterior/posterior cingulate cortices and unilaterally in left medial prefrontal, right orbitofrontal, left superior temporal, right middle/inferior temporal, right insular, left hippocampal, right parahippocampal, and right lateral occipital cortices, and in the left cuneus, thalamus, and cerebellum
Lin *et al*.^23^	21 (85.71)	15 (73.33)	40.1(10.8)	39.8 (9.53)	26.24 (3.40)	38.77 (19.91)	26.59 (19.38)	NA	FWE corrected, *p* < 0.05	GMV↑ in right insular; GMV↓ in left anterior cingulate gyrus

GMD, gray matter density; GMV, gray matter volume; FWE, family-wise error; FDR, false discovery rate; NA, not available; OSA, obstructive sleep apnea; HC, healthy control; BMI, body mass index; AHI, apnea–hypopnea index; ODI, oxyhemoglobin desaturation index; ESS, Epworth sleepiness scale; SD, standard deviation; SVC, small volume correction.

^*^VBM analysis based on 25 patients and 23 controls; ^a^sham (subtherapeutic) OSA patients; ^b^active (therapeutic) OSA patients.

### VBM meta-analysis

**Figure 2:**
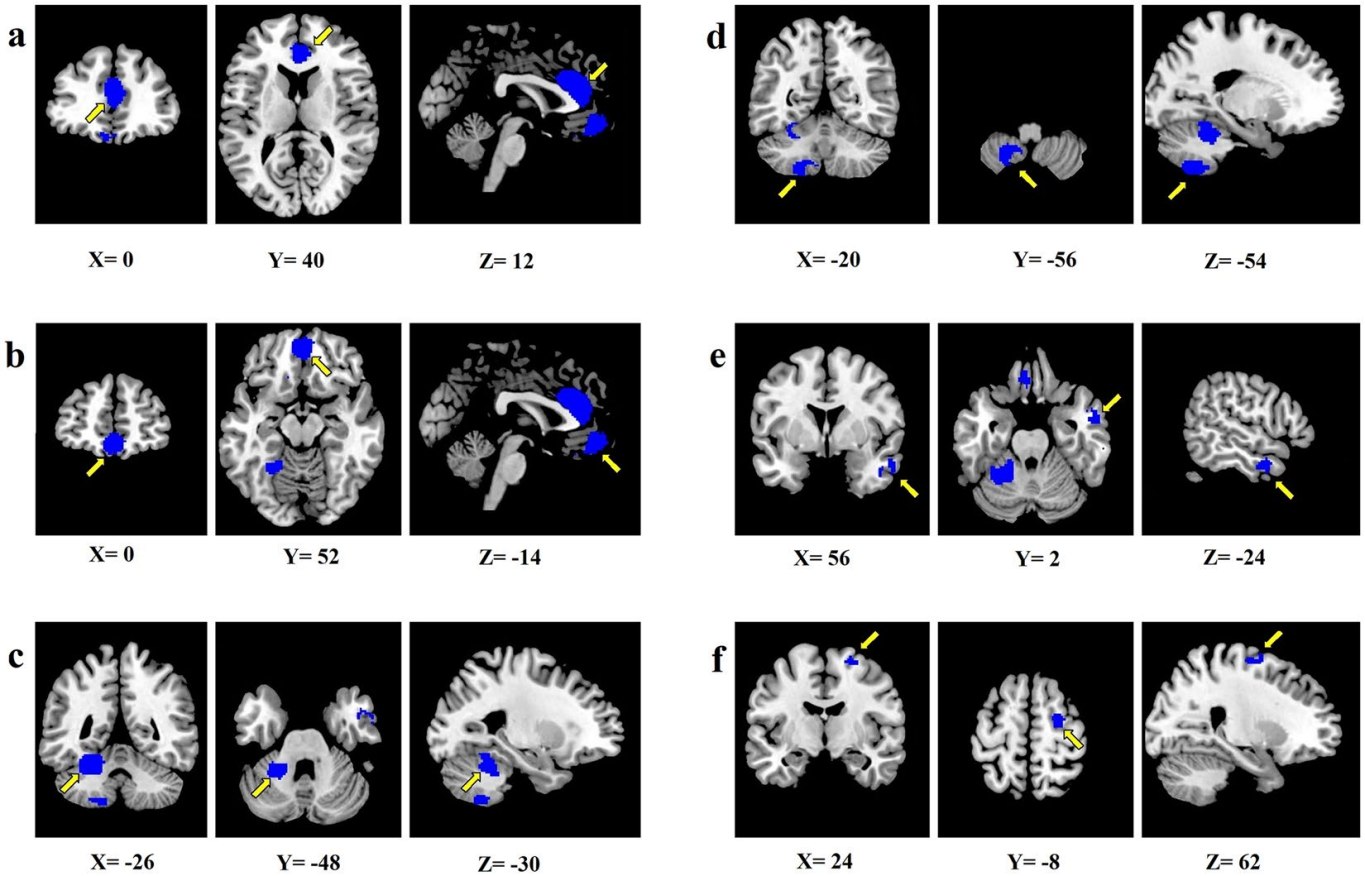
The meta-analysis revealed six brain regions showing reduced gray matter in patients with OSA compared with that in healthy controls. (**a**) Bilateral ACG/ApCG; (**b**) Bilateral SFG (medial rostral part); (**c**) Left cerebellum, hemispheric lobules IV/V; (**d**) Left cerebellum, hemispheric lobule VIII; (**e**) Right MTG; (**f**) right premotor cortex. Abbreviations: ACG/ApCG, anterior cingulate/paracingulate gyri; MTG, middle temporal gyrus; SFG, superior frontal gyrus.

The meta-analysis revealed six brain regions with GM reductions in patients with OSA relative to healthy controls: (1) bilateral anterior cingulate/paracingulate gyri (ACG/ApCG, one cluster involving both brain sides); (2) bilateral superior frontal gyrus (SFG, medial rostral part) (one cluster involving both brain sides); (3) left cerebellum, hemispheric lobules IV/V; (4) left cerebellum, hemispheric lobule VIII; (5) right middle temporal gyrus (MTG); and (6) right premotor cortex (Table 2 and Fig. 2).

**Table 2:** Regional gray matter reductions in patients with OSA compared with that in healthy controls identified by meta-analysis.

**Region**	**MNI coordinates**			**SDM** ***z*** **score**	***p*** **, uncorrected**	**Voxels**	**Cluster breakdown (voxels)**
	x	y	z				
1. Bilateral anterior cingulate/paracingulate gyri	0	40	12	−2.367	0.000335693	968	Left anterior cingulate/paracingulate gyri (539)
							Right anterior cingulate/paracingulate gyri (367)
							Left superior frontal gyrus, medial, BA 32 (62)
2. Bilateral superior frontal gyrus (medial rostral part)	0	52	−14	−2.371	0.000331044	614	Left superior frontal gyrus, medial rostral (254)
							Right superior frontal gyrus, medial rostral (159)
							Left gyrus rectus (143)
							Right gyrus rectus (58)
3. Left cerebellum, hemispheric lobules IV/V	−26	−48	−30	−2.064	0.001512885	561	Left cerebellum, hemispheric lobules IV/V (214)
							Left cerebellum, hemispheric lobule VI (185)
							Left fusiform gyrus (119)
							Middle cerebellar peduncles (43)
4. Left cerebellum, hemispheric lobule VIII	−20	−56	−54	−2.193	0.000813842	333	Left cerebellum, hemispheric lobule VIII (234)
							Left cerebellum, hemispheric lobule IX (99)
5. Right middle temporal gyrus	56	2	−24	−2.178	0.000878394	136	Right middle temporal gyrus (116)
							Right temporal pole, middle temporal gyrus (20)
6. Right premotor cortex	24	−8	62	−2.006	0.001998782	103	Right premotor cortex, BA 6 (103)

Voxel-wise *p* < 0.005. HC, healthy control; MNI, Montreal Neurological Institute; OSA, obstructive sleep apnea; SDM, signed differential mapping.

### Sensitivity analysis

The whole-brain jack-knife sensitivity analysis revealed that GM reductions in the bilateral ACG/ApCG and bilateral SFG (medial rostral part) were highly reliable and preserved throughout all 16 dataset combinations. The results in the left cerebellum, hemispheric lobule VIII were preserved in 15 dataset combinations. The other clusters failed to be repeated in two or three combinations, especially when the studies by Joo *et al*.^21^, Kim *et al*.^18^ and Morrell *et al*.^31^ were excluded (Table 3).

**Table 3:** Jack-knife sensitivity analyses of gray matter reductions in the pooled meta-analysis

Studies excluded	Canessa	Celle	O’Donoghue	Fatouleh	Huynh -1	Huynh -2	Innes	Joo	Kim	Lin	Morrell^a^	Morrell^b^	Morrell^c^	Torelli	Yaouhi	Zhang	Rank	Male only	Middle- aged	EDS
1. Bilateral anterior cingulate/paracingulate gyri	Y	Y	Y	Y	Y	Y	Y	Y	Y	Y	Y	Y	Y	Y	Y	Y	1	Y	Y	Y
2. Bilateral superior frontal gyrus, medial rostral	Y	Y	Y	Y	Y	Y	Y	Y	Y	Y	Y	Y	Y	Y	Y	Y	1	Y	Y	Y
3. Left cerebellar lobules IV/V	Y	Y	Y	Y	Y	Y	Y	N	N	Y	Y	N	Y	Y	Y	Y	4	Y	Y	Y
4. Left cerebellar lobule VIII	Y	Y	Y	Y	Y	Y	Y	Y	Y	Y	Y	Y	N	Y	Y	Y	2	N	Y	Y
5. Right middle temporal gyrus	Y	Y	Y	Y	Y	Y	Y	N	Y	Y	Y	Y	N	Y	Y	Y	3	N	Y	Y
6. Right premotor cortex	N	Y	Y	Y	Y	Y	Y	N	N	Y	Y	Y	Y	Y	Y	Y	4	Y	Y	Y

Y, the cluster was repeated; N, the cluster was not repeated.

### Subgroup analysis

In the male-only subgroup analysis of 9 datasets, the results remained largely unchanged. Compared with the pooled analysis of males and females, all clusters remained except two regions: the left cerebellum (lobule VIII) and right MTG (Table 3).

The results of the middle-aged subgroup (containing 15 datasets) and EDS subgroup (containing 9 datasets) analyses were both identical to the results of the pooled analysis (Table 3).

### Meta-regression analysis

**Figure 3:**
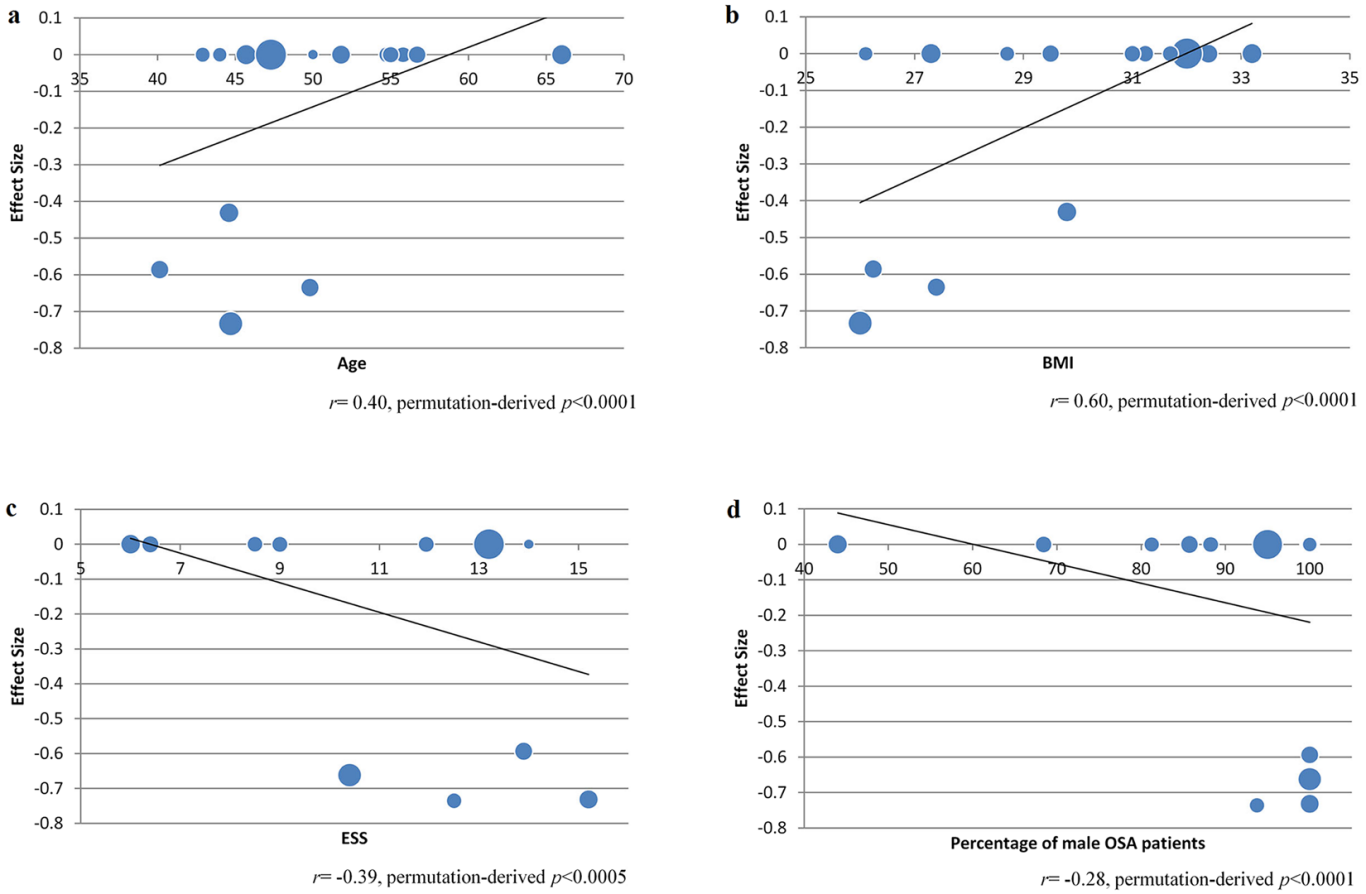
Meta-regression results showed that the mean age (**a**) and BMI (**b**) of patients with OSA were positively correlated with gray matter in the bilateral ACG/ApCG and that the ESS scores of patients with OSA (**c**) and the percentages of male patients with OSA (**d**) were negatively correlated with gray matter in the bilateral SFG (medial rostral part). The effect sizes in this graph were extracted from the peak of maximum slope significance, and the dot size reflects the sample size of each study. Abbreviations: ACG/ApCG, anterior cingulate/paracingulate gyri; SFG, superior frontal gyrus.

The GM in the cluster of bilateral ACG/ApCG was positively associated with age (*r* = 0.40, permutation-derived *p* < 0.0001, Fig. 3a) and BMI among patients with OSA (*r* = 0.60, permutation-derived *p* < 0.0001, Fig. 3b), and both relationships were primarily observed in four studies^18, 21, 23, 43^. The GM in the bilateral SFG (medial rostral part) was negatively associated with ESS scores (*r* = −0.39, permutation-derived *p* < 0.0005, Fig. 3c) and the percentage of male patients among patients with OSA (*r* = −0.28, permutation-derived *p* < 0.0001, Fig. 3d); these results were also primarily observed in four studies^18, 21, 30, 43^. No significant linear relationships were observed between neuroanatomic measures and the AHI or ODI scores.

## Discussion

Our study identified consistent and robust regional GM alterations in patients with OSA compared with healthy controls. Additionally, to the best of our knowledge, this meta-analysis is the first to explore the potential effects of age, sex and important clinical characteristics on GM alterations due to OSA. Our main finding was that patients with OSA exhibited reduced GM in bilateral ACG/ApCG, bilateral SFG (medial rostral part), left cerebellum (hemispheric lobules IV/V and VIII), right MTG and right premotor cortex. These results partially overlapped with those of two previous meta-analyses, particularly the meta-analysis by Wang *et al*.^47^ (regions of the frontal and temporal lobes). Differences mainly existed regarding the cerebellum and some subcortical structures, which might have been detected given the larger number of studies included in our analysis. Our results remained similar when the analyses were repeated with the subgroups of male subjects, middle-aged subjects and subjects who scored equal or greater than 10 on the ESS scale, which supports the robustness of our results. Furthermore, the GM values from brain regions with GM alterations were significantly correlated with mean age, BMI, ESS score and percentage of male patients in the OSA group, suggesting that these GM abnormalities may be associated with these clinical factors.

### Findings in the anterior cingulate/paracingulate gyri

The anterior cingulate cortex (ACC) is part of the limbic system, which is a crucial structure in humans that participates in the control of various autonomic, cognitive and emotional functions^52^. Although recent accounts of ACC function have placed more emphasis on its role in cognitive and emotional processes, other research has clearly established that the ACC is involved in central cardiovascular and respiratory control^53^. Respiratory arousal has been positively correlated with ACC thinning^54^, and response signals were altered in the ACC during Valsalva maneuvers and expiratory loading tasks in patients with OSA^40, 41^. GM reductions in the cingulate cortex have been consistently reported in patients with OSA^18, 21, 23, 27^. In one of our included VBM studies, GMV within the ACC in OSA patients was reduced before and after multi-level surgical treatment compared with that in controls, suggesting that the reduced GMV of this structure might be an irreversible deficit following OSA^23^. The ACC participates in attention and executive processes^55^, and an atrophic ACC may partially explain the cognitive deficits in patients with OSA. From a network perspective, the dorsal part of the ACC is a key node in the salience network (SN), which is involved in detecting, integrating and filtering relevant interoceptive, autonomic and emotional information^56^. The insula, which has been reported to be abnormal in OSA, is another key node in the SN. Several structural studies have demonstrated GM alternations in the insula in patients with OSA. However, the results have not been concordant across studies. For example, the insula has been reported to exhibit both significant GM atrophy^18, 21^ and enlargement^22, 23^. This evidence indicates a potential role of the SN in OSA. The paracingulate gyrus is adjacent to the cingulate gyrus, located at the border of Brodmann areas (BAs) 32 and 10. The exact function of this structure is less well understood, but the paracingulate gyrus has been related to the understanding of intentions during social interactions^57^. Further work is needed to explore this structure in OSA.

Although the current meta-analysis did not reveal alterations in other components of the limbic system, it is noteworthy that the hippocampus, parahippocampus and amygdala are three limbic structures commonly found to be abnormal in OSA studies, as reported in some of our included VBM studies^16, 18, 21, 29, 30, 32, 49^. The previous meta-analysis conducted by Tahmasian *et al*.^33^ revealed structural atrophy and functional disturbances in the right basolateral amygdala/hippocampus and the right central insula, suggesting an impact of OSA on these structures that might contribute to the cognitive and affective alterations seen in patients with the disorder. The “non-significance” of these three structures in the current meta-analysis was in part due to contradictory findings, e.g., the report by Fatouleh *et al*.^22^ of increased GMV in the left hippocampus.

### Findings in the frontal lobe

In the current study, GM reductions were observed in two parts of the frontal lobe, including the medial rostral part of the SFG bilaterally and the right premotor cortex. Alteration in the SFG’s rostral and ventral extension to the orbital frontal cortex (OFC) suggests an important contribution of this region to the neurobehavioral aspects of OSA. The OFC is the ventromedial part of the frontal lobe and shares extensive connections with other parts of the prefrontal cortex, with the limbic, motor, premotor, sensory and association cortices, and with multiple subcortical regions^58^. This region of the ventromedial prefrontal cortex (VMPFC) appears vulnerable to sleep loss. For example, total overnight sleep deprivation was associated with reductions in cerebral glucose metabolism in the prefrontal cortex, which appeared to be particularly prominent in the VMPFC^59^. The OFC is a critical structure in the nervous system that facilitates cognitive functions and the integration of cognitive and emotional processes guiding decision-making^60^. According to behavioral data, sleep loss leads to impaired decision-making, increased risk-taking behaviors^61^ and emotional difficulties^62^. It is of note that the medial OFC is a component of the default mode network (DMN), which is typically more active during rest than during cognitive activity. Within this network, altered functional connectivity and regional brain activity were observed in the resting state^63^, and abnormal deactivation was observed during working memory tasks in patients with OSA^36^. Thus, the GM atrophy of the OFC identified in the current study may represent a brain change resulting from low sleep quality and hypoxia during sleep, leading to the cognitive deficits and emotional problems seen in patients with OSA.

Another region of GM alteration in the frontal lobe was located in right premotor cortex. In healthy humans, the right premotor cortex is important in voluntary behavior, including volitional inspiration^64^ and voluntary breathing^65^. However, in the apnea state, the functional activation of the premotor cortex is altered bilaterally^66^. The function of the premotor cortex is not limited to the motor domain; it is also active during executive cognitive processes^67, 68^. Sleep loss has also been shown to affect the functional activation in the premotor cortex during finger motor tasks^69^. Among patients with OSA, structural studies have demonstrated GM atrophy bilaterally^21^, and one study reported increased GMV in the left premotor cortex^22^.

### Findings in the middle temporal lobe

The MTG is located on the lateral surface of the temporal lobe and is involved in multiple cognitive processes^70^. One tractography-based parcellation study suggested that the anterior subregion of the MTG is primarily involved in the DMN, sound recognition, and semantic retrieval; that the middle subregion is predominantly involved in semantic memory and semantic control networks; and that the posterior subregion is important for language. The sulcus defining the posterior MTG is associated with coding gaze and perception of motion in the visual field^71^. Structural and functional deficits in this region have been reported in epilepsy^72^, schizophrenia^73^ and major depression^74^. Many structural studies have reported GM reductions in the temporal lobe in patients with OSA^18, 21, 27, 30, 31^. According to functional studies, altered cerebral activation in the temporal cortex is significantly related to nocturnal hypoxemia duration (hemoglobin oxygen saturation levels < 90% during sleep) in OSA patients^36, 75^, and nocturnal hypoxemia is thought to be important in mediating cardiovascular damage^76^. In our study, the anterior part of the MTG, which is adjacent to the right temporal pole, was the region most consistently altered in OSA across different VBM studies. This finding overlaps with the results of the meta-analysis of studies on major depression conducted by Zhang *et al*.^74^ and is consistent with the view that alterations in this region may also contribute to emotional and cognitive changes in OSA. At the network level, the anterior MTG is traversed by abundant white matter tracts^77^, including the uncinate fasciculus, arcuate fasciculus, fornix, anterior commissure and inferior longitudinal fasciculus, which participate in different brain networks. For example, a resting-state fMRI study found that the anterior MTG exhibited positive connectivity with other components of the DMN, including the hippocampus, medial superior frontal cortex, and posterior cingulate cortex, as well as negative connectivity with frontotemporal brain areas^71^. Thus, deficits in this region may cause undesirable impacts on both intrinsic and integrated communications from the DMN, which would represent a significant alteration in resting brain neurophysiology.

### Findings in the cerebellum

Findings in the left cerebellum also indicate the involvement of a motor-related network along with a cluster in the premotor cortex. Although the cerebellum is a crucial module in motor control systems, it is also connected to widespread brain regions, including cortical association areas and limbic structures^78^. Via these connections, the cerebellum participates in respiratory modulation^79^, sensorimotor activities, motor learning, emotional functions^80^, and even social functions^81^. The cerebellum is also important for maintaining sleep^82^, and sleep loss may disturb cerebellar function. Sleep deprivation disrupts fMRI signals from many brain regions, including the cerebellum during sensorimotor tasks^83^. Most neuroimaging studies have reported decreased GMV^18, 20, 21, 30, 31^ and elevated neural activation of the cerebellum in patients with OSA^38, 84^ compared with those in healthy controls, despite some inconsistencies^22^. Because the cerebellum is sensitive to carbon dioxide^85^ and because there is a negative correlation between cerebellar GMV and AHI or ODI^20^, our results might be associated with repetitive intermittent hypoxia or disturbed nocturnal sleep. According to our results, GM atrophy occurs in cerebellar hemisphere lobules IV, V, VI, VIII, and IX. Lobule VI is a crucial node in sensorimotor control^86^. Additionally, strong resting-state functional connectivity exists among different lobules and various brain networks, such as among lobule IX, vermal VIIIb and the DMN and among lobules VI, VIIb, and VIII and the SN^87^. Thus, the cerebellum is extremely important for the proper functioning of both motor and cognitive networks, and its alteration may contribute to behavioral changes associated with OSA.

A comparison of the findings of our study with those of two previous meta-analyses reveals partial overlap, indicating the involvement of the DMN, SN and limbic system in OSA. Therefore, our findings provide support for the involvement of these brain systems in OSA from a network perspective. Moreover, a prior review on OSA have highlighted that sleep disturbance and hypoxia could not explain all impacts on brain systems in ways that may contribute to cognitive deficits^88^. Our findings expand those observations by highlighting additional disturbances in brain regions that support affect processing. For example, while a recent review emphasized the importance of posterior DMN in OSA^89^, our review that includes more recently published studies highlights the importance of anterior DMN regions, which are regions more important for emotion modulation and motivation than sensory/perceptual processing.

### Effects of demographic variation in GM changes

ESS scores were negatively correlated with GM in the bilateral OFC (patients who had higher ESS scores tended to have smaller GM values in the OFC). Additionally, the results of the EDS subgroup analysis were identical to those of the pooled analysis. The ESS score subjectively measures the severity of OSA and reflects the degree of daytime sleepiness with some limitations. Daytime sleepiness is a consequence of sleep loss during the night, and sleep loss *per se* may lead to abnormal brain activity in the ACC^90^, OFC^91^, and cerebellum^92^ or GM volume reductions in the cerebellum and MTG^93^. These abnormalities may consequently cause the cognitive and affective problems observed in patients with OSA. For example, sleepiness could predict vigilance performance in patients with OSA^94^, and the severity of EDS predicted incident depression in the patients^95^.

Interestingly, no correlation was found between AHI or ODI and GM changes. Previous VBM studies reported significant negative^16, 20^, positive^19, 23^ or no^43^ correlations between AHI/ODI and GMV in different brain areas. These findings suggest that the integrated influence of these divergent findings may have been balanced out in the meta-analysis, thus revealing no consistent pattern of effect. The present results are also in accordance with the viewpoint that both AHI and ODI can only partly explain the phenotypes of OSA^96^. While these scales are important for evaluating acute illness severity over recent days or weeks, they may not accurately reflect the severity of sleeplessness and hypoxia over months and years of OSA, which may be the primary cause of GM alterations.

In addition, although no study included in our meta-analysis used RDI, this index extends the notion of AHI by emphasizing the role of RERA, a condition of arousal from sleep caused by increased respiratory effort seen in some OSA patients. To our best of our knowledge, only one VBM study used both AHI (34 ± 20 events/h) and RDI (38 ± 24 events/h) to define OSA patients; however, the authors of that study did not perform a correlation analysis to examine the relationships between these two indexes and GMV changes^27^. Another study exploring the effect of obesity on brain diffusion found that both RDI and AHI showed positive correlations with the apparent diffusion coefficient (ADC) in several brain areas, although RDI showed a more extensive impact, suggesting that RDI might be more sensitive than AHI to microstructural changes^97^. Considering this finding, despite some debates regarding the role of RERA in OSA, future meta-analyses might benefit from comparing OSA patients with and without RERA once more studies using RDI are published. Another area where greater work is needed is the effort to resolve the long-standing debate about the relative importance and differential impacts of sleep apnea and sleep fragmentation in causing GM changes in patients with OSA.

Our regression analysis revealed a positive correlation between the age of the patient group and GM in the bilateral ACG. In prior OSA studies, the correlations between age and GMV were not consistent. Macey *et al*.^27^ reported that age was significantly and negatively correlated with the total GMV in controls but not in the patients. Torelli *et al*.^32^ reported a negative correlation between age and GMV in the hippocampus/amygdala in patients with OSA. One study reported a negative result^21^. Our regression analysis indicated that older patients tended to have less GM atrophy in the ACG. This finding may be related to the GM pattern changes that occur during normal aging. As most of the OSA patients included in our meta-analysis were middle-aged, the age range was much smaller than typically used in studies comparing age effects, and age may be confounded with illness duration, age associations raise issues that need to be addressed in longitudinal studies.

The BMI of patients with OSA was also positively associated with GM in the bilateral ACG. Obesity is related to OSA and sleep loss. Regarding the relationship between sleep loss and BMI, sleep loss can affect the connections in the SN when accompanied by a higher intake of dietary fat^98^. One recent study using a cohort of 1,333 OSA patients found that more than 80% of patients were overweight or obese, and obesity was a more important factor in contributing to OSA than sex (male)^15^. Although our regression analysis suggested a positive relationship between BMI and GMV among OSA patients, in another study, obese (BMI > 30) subjects (but not overweight (BMI = 25–30) subjects) exhibited GM atrophy in the ACG and OFC compared with normal-BMI subjects^99^. Therefore, the influence of body fat on brain GM should be clarified in future studies.

OSA is more prevalent among males and is likely more severe among males^100^. Our subgroup analysis of studies including only male participants yielded results similar to the analysis of male and female patients combined, except for two clusters (left cerebellum (lobule VIII) and right MTG) that were no longer significant in the male-only analysis. This result might be due to the smaller sample of patients, or it may suggest some sex differences in the brain impact of OSA. For example, the percentage of male patients was negatively correlated with GM in the OFC, indicating that a greater proportion of males in the patient group resulted in more GM atrophy in this region. This finding again underscores the importance of sex differences in OSA.

The above findings regarding demographic effects raise questions regarding the relevance of OSA phenotypes, which have largely been ignored by individual VBM studies but are more readily examined via meta-analyses. As there is still no consensus regarding standardized diagnostic criteria for OSA, with AHI cut-off criteria considered an interim standard^101^, many researchers regard OSA as a heterogeneous disease in terms of symptoms, etiology, comorbidities and outcomes. At present, factors including AHI, EDS, sex, age, obesity and race/ethnicity are all recognized as phenotypes or important factors impacting OSA^102, 103^. Our study demonstrated that these phenotypes based on clinical or demographic information had both significant (e.g., age and sex) and non-significant (e.g., AHI and ODI) relationships with GMV. The lack of relationships between GM measurements and current and recent clinical features of OSA raises the possibility that cumulative long-term effects, which appear variable in relation to demographic factors, may impact brain anatomy in a way that single-point assessments fail to reflect, especially for scales that rate clinical features during only the most recent few days.

Given the effects we observed, MRI may be suitable for clinical evaluations of OSA, especially for patients showing neuropsychiatric symptoms. Such changes may indicate a need for more aggressive therapy or support for therapeutic compliance to prevent further adverse brain changes that could significantly reduce quality of life and hopefully to reverse some abnormalities.

## Limitations and Conclusions

Our study has some limitations. First, this meta-analysis was coordinate-based, and this approach has some inherent inaccuracies compared with image-based meta-analyses. Second, the results may be biased because different studies use different statistical thresholds and different criteria for OSA diagnoses. Third, the jack-knife sensitivity analysis revealed that the findings of GM reductions in the right premotor cortex, left cerebellar lobules and right MTG were less robust than alterations in other brain regions; thus, these findings should be interpreted with caution. Fourth, although most researchers carefully excluded comorbid diseases or sleep disorders and treatment history, participant heterogeneity remained that may have biased the results, such as those regarding the severity, duration and cause of OSA; the degree of nocturnal desaturation; the presence of predominant apneas or hypopneas; and sleep quality. Fifth, the small cohorts of some of the included studies might have biased the results.

Taken together, we identified GM reductions in the limbic lobe, frontal lobe, temporal lobe, and cerebellum in OSA patients compared with those in healthy controls. The most reliable findings were seen in the bilateral ACG/ApCG and bilateral SFG (medial rostral part). Additionally, the age, BMI, ESS, and sex (male) of the patient group were associated with GM atrophy. The intra- and inter-networks of DMN, SN, and motor networks and the limbic system were observed in OSA. Further exploration is required to verify our conclusions through multimodality neuroimaging studies and longitudinal studies and to determine their clinical relevance for clinical practice in the diagnosis and management of patients with OSA. Of note, this study adds to Psychoradiology (https://radiopaedia.org/articles/psychoradiology), an emerging subspecialty of radiology, which seems primed to play a major clinical role in guiding diagnostic and treatment planning decisions in patients with mental disorder^104, 105^.
